# An intervention to improve care and reduce costs for high-risk patients with frequent hospital admissions: a pilot study

**DOI:** 10.1186/1472-6963-11-270

**Published:** 2011-10-13

**Authors:** Maria C Raven, Kelly M Doran, Shannon Kostrowski, Colleen C Gillespie, Brian D Elbel

**Affiliations:** 1 Department of Emergency Medicine, NYU School of Medicine, 462 First Avenue, New York, NY 10016, USA; 2 Department of Emergency Medicine, UCSF School of Medicine, 505 Parnassus Ave, San Francisco, CA 94143, USA; 3 Robert Wood Johnson Foundation Clinical Scholars Program, Yale University School of Medicine and U.S. Department of Veterans Affairs, USA; 4 Division of General Internal Medicine, NYU School of Medicine, 423 East 23rd Street, #15028AN New York, NY 10016, USA

## Abstract

**Background:**

A small percentage of high-risk patients accounts for a large proportion of Medicaid spending in the United States, which has become an urgent policy issue. Our objective was to pilot a novel patient-centered intervention for high-risk patients with frequent hospital admissions to determine its potential to improve care and reduce costs.

**Methods:**

Community and hospital-based care management and coordination intervention with pre-post analysis of health care utilization. We enrolled Medicaid fee-for-service patients aged 18-64 who were admitted to an urban public hospital and identified as being at high risk for hospital readmission by a validated predictive algorithm. Enrolled patients were evaluated using qualitative and quantitative interview techniques to identify needs such as transportation to/advocacy during medical appointments, mental health/substance use treatment, and home visits. A community housing partner initiated housing applications in-hospital for homeless patients. Care managers facilitated appropriate discharge plans then worked closely with patients in the community using a harm reduction approach.

**Results:**

Nineteen patients were enrolled; all were male, 18/19 were substance users, and 17/19 were homeless. Patients had a total of 64 inpatient admissions in the 12 months before the intervention, versus 40 in the following 12 months, a 37.5% reduction. Most patients (73.3%) had fewer inpatient admissions in the year after the intervention compared to the prior year. Overall ED visits also decreased after study enrollment, while outpatient clinic visits increased. Yearly study hospital Medicaid reimbursements fell an average of $16,383 per patient.

**Conclusions:**

A pilot intervention for high-cost patients shows promising results for health services usage. We are currently expanding our model to serve more patients at additional hospitals to see if the pilot's success can be replicated.

**Trial registration:**

Clinicaltrials.gov Identifier: NCT01292096

## Background

A small percentage of Medicaid patients, many of whom are affected by multiple chronic diseases including mental illness and substance use, account for a disproportionate share of emergency department (ED) and inpatient visits and costs [1,2]. Overall, 4% of Medicaid patients account for nearly half of Medicaid spending, around $88 billion in 2001 [3]. These high-cost cases have caught the attention of policy makers, and many state leaders are focusing on this small group of highest-cost beneficiaries as a way to bend the cost curve and improve quality of care [4]. All of these factors present an imperative for developing successful models to provide cost-effective care for the highest users of health services.

Several interventions targeting high-risk patients in Medicaid and other arenas have demonstrated success in controlling costs while decreasing frequent use of emergency department (ED) and inpatient services. A recent randomized control trial in Chicago showed that providing housing and case management to homeless adults with chronic conditions reduced future hospital days and ED visits [5]. Another Chicago study showed that providing respite care (twenty-four hour room and board along with social services) to homeless patients after hospital discharge reduced future hospitalizations [6]. A randomized trial aimed at frequent ED users in San Francisco showed a reduction in ED visits with case management, but no significant decrease in hospital admissions [7]. Several other studies have shown success in reducing both hospitalizations and costs with case management plans directed toward seriously mentally ill patients and the elderly [8-10].

These prior studies had variable patient selection criteria, none of which were based on validated estimates of predicted future costs and health services utilization. Thus, limited intervention resources may have been misdirected towards patients whose hospital admissions may have decreased even without intervention. Interventions have the most potential for cost savings when they are directed specifically toward those who will in the future be the most frequent users of expensive health services. For example, one analysis of a care coordination program for Medicare beneficiaries revealed that the entirety of the program's cost savings and reduction in hospitalizations was accounted for by the subset of highest severity cases [11].

Focusing on patients with frequent hospitalizations is of consequence because hospital admissions make up a substantial portion of Medicaid expenditures, whereas reimbursements for other services, such as ED and outpatient visits, are much lower. Previous work has developed and validated a regression algorithm that uses hospital administrative data to identify Medicaid patients who are at high risk of subsequent re-hospitalization in the following 12 months based on their diagnoses and service utilization history in the prior 3 years [12,13]. Using this case-finding algorithm allows us to identify patients in real-time who stand to benefit the most from a comprehensive care management intervention. In prior research we observed several remediable risks in patients identified by the case-finding algorithm [13]. The majority of patients had no usual source of care or identified the ED as their usual source of care. In addition, over half were homeless or precariously housed, and most had little family or social support. Substance use and mental illness were more prevalent than other chronic illness. Via qualitative interviews, it was clear that all of these factors affected patients' health services use.

The current study describes a pilot intervention at one New York City public hospital for Medicaid patients identified by the validated case-finding algorithm as being at high risk of future hospital admissions. The intervention was designed to improve care and reduce costly hospital admissions via a patient-centered intensive care management program. The aim of the pilot was to ensure that the needed hospital and community services could be delivered to high-risk patients in a coordinated and patient-centered manner. We also aimed to conduct a preliminary analysis of the program's impact on health services use and costs. Recognizing that patients with frequent use of health services have become an urgent policy issue, our objective is to describe in detail our pilot intervention and its initial outcomes, in hopes that others might be able to use this timely information to design their own similar programs.

## Methods

### Population studied

#### Study setting

The current study took place at Bellevue Hospital Center (BHC), an 809-bed public hospital in New York City that serves as a "safety net" hospital for a diverse and primarily underserved population, with 500,000 outpatient visits and over 100,000 ED visits yearly [14].

#### Algorithmic case-finding

We used a previously validated, predictive case-finding algorithm to identify patients at high risk for future hospital admissions, described in detail elsewhere [1,12,13]. In brief, the algorithm can employ hospital or State level administrative data to identify patients who are at high risk of subsequent readmission in the next 12 months based on their ICD-9 diagnoses and service utilization history (e.g., ED visits, inpatient admissions, outpatient clinic visits) over the past four to five years. In the current study, hospital level data from the previous five years was used. Patients were identified for inclusion in real time as they were admitted to the hospital, and could be approached for enrollment either in the ED or the inpatient setting. The algorithm assigns each patient a risk score of 0 to 100, with 100 being those patients at highest risk of subsequent readmission in the following 12 months. By employing this predictive model we were able to target specifically those patients predicted to be high risk in the next 12 months and stood to benefit from an intervention, rather than patients who may be heavy users today, but whose use patterns will regress to the mean without intervention (the majority). We chose a cut-off of greater than 50 as a criterion for enrollment based on the high probability (positive predictive value 0.7) of hospital admission in the following 12 months for this score [13].

#### Patient enrollment

Eligible patients met the following criteria: Medicaid fee-for-service patient aged 18-64; current admission into any inpatient unit at BHC; an algorithm risk score for readmission of greater than or equal to 50; and the ability to speak either English or Spanish. Dual-eligible patients (those covered by both Medicaid and Medicare) were excluded. We enrolled patients from November 2007 through March 2008. On weekdays we conducted a computer query to ascertain whether any patients admitted to the hospital in the preceding 24 hours met eligibility criteria; on Mondays this query included all patients admitted over the weekend. Upon identification, eligible patients were approached for enrollment if they were still in the hospital. Patients institutionalized in nursing homes or prisons prior to their admission were excluded based on the fact that they have important factors affecting their hospitalizations that differ from the non-institutionalized population [15]. Consecutive patients eligible for inclusion were approached for enrollment and consented during their index hospital admissions. Our initial enrollment target was 15 patients, which we felt would be sufficient to test the feasibility of our pilot intervention. To achieve this with the expected loss to follow up, we eventually enrolled a total of 19 patients.

The study was approved by the Institutional Review Boards of the New York University School of Medicine and Bellevue Hospital Center.

### Intervention

Using knowledge gained from other programs throughout the country and from our previous in-depth qualitative interviews with 50 high-risk patients and their in-hospital providers at Bellevue Hospital Center (BHC), we identified the following key principles that would guide our intervention [13]:

1. Care must be coordinated and responsive to specific patient needs.

2. Care must not end at hospital discharge, but continue into the community.

3. Medical homes and permanent housing are essential.

4. Integrated, multidisciplinary services and provider teams are necessary to care for the whole patient.

5. Care teams must serve patients where they are, both physically and mentally.

6. Data sharing and adequate communication among team members is essential for care coordination and tracking patients' progress.

Based on these principles we designed an intervention that was patient-centered, with intensive and flexible case management tailored to the individual. Key operating guidelines included the use of a harm reduction model (no sobriety required for participation in the program) and a multidisciplinary team approach. From previous pilot research we had learned that in addition to having complex medical needs, our high-risk patients would also have complex social needs [13]. Therefore, our intervention extended beyond hospital doors into the community and was conducted in partnership with community providers of homeless, mental health, substance use, and other key services.

A multidisciplinary intervention team was based at BHC. Most patient contact was via a "Community-Based Care Manager" responsible for providing care management and coordination both inside and outside the hospital system. Care Managers were required to have a minimum of a high school education and requisite experience working with the type of high-risk population targeted for intervention. A master's level project director assisted with patient interviews, consents, and administrative responsibilities. A master's level social worker was responsible for the clinical biopsychosocial needs assessments for enrolled patients and helped coordinate patients' discharge plans at their index hospitalizations. The physician PI of the pilot (MR) oversaw the project and intervention team.

The intervention began at the patient's bedside during the enrollment hospitalization. Patients underwent in-depth interviews to identify immediate and long-term needs such as housing, primary care, transportation to and advocacy during appointments, medication management, entitlements enrollment, improved connections to psychiatric and substance use treatment, and home visits. Study staff worked closely with inpatient providers to facilitate appropriate discharge planning and follow-up. No patients were discharged to the street or a shelter. Patients who met criteria for chronic homelessness were evaluated in-hospital by a community housing partner, the Common Ground Community, who initiated housing applications for permanent housing. If a waiting period was necessary, we offered patients discharge into "stabilization" housing at the YMCA until their applications for permanent housing were complete. We used a Housing First model (sobriety not a prerequisite for housing), which has shown success in other published interventions [16,17].

Services continued after hospital discharge into the community and were tailored to the needs of each patient. The Community-Based Care Manager facilitated transportation to appointments, assisted with entitlements enrollment, conducted home visits, and connected patients to other needed medical and non-medical services. Pre-paid cellular phones were provided to patients to allow close contact with study staff for reminder calls and crisis management. Patients were provided with expedited medical appointments through cooperation with the BHC outpatient clinics, and Care Managers would accompany patients and advocate for them during appointments when necessary. For patients in the ED, Care Managers assisted ED staff by providing collateral information and helping to ensure follow-up for enrolled patients who were treated and released. Reminder calls for appointments were needed for most patients.

One key to our intervention was close communication with various service providers relevant to the patients in the community. For example, we had regular contact with Visiting Nurse Services, methadone programs, substance abuse rehabilitation centers, and outpatient clinics involved with our patients. Weekly conference calls with relevant providers allowed everyone to be "on the same page" regarding patients' care plans. Conference calls were limited to one hour and held at a regularly scheduled time each week with participants present in person whenever possible. At a minimum, participation occurred from the core team consisting of the Care Manager, Social Work Supervisor, and housing partner who could relay input regarding the patient's care plan as appropriate. Others (primary care physicians, psychiatrists) who were not directly part of the intervention team would participate as needed and based on availability. Therefore, when patients presented to the ED or were admitted to the hospital our intervention staff could assist their care providers in formulating appropriate patient management and discharge plans.

### Data collection

Patients were interviewed at study enrollment to obtain demographic, health status, and behavioral health information. Although this study was designed primarily to test intervention feasibility, we also collected objective measures of pilot effectiveness from our hospital's administrative database, including numbers of hospitalizations, ED visits, and clinic visits, and examined Medicaid billing and reimbursement data for every patient admission and visit to the clinics or ED. Care managers were asked to track all contacts with project participants to analyze staff time allocations and activities. Univariate statistics (means and frequencies) were derived from the quantitative data. In addition, paired-sample t-tests were used to compare differences between visits and costs before and after study enrollment.

In addition, care managers and our direct patient services staff employed by our community housing partner, Common Ground Community, participated in a focus group led by research staff trained in qualitative methods (MR and CG). The qualitative interview instrument contained semi-structured questions that allowed those caring for enrolled patients to discuss challenges of working with the enrolled population, health system barriers to coordinating their care, and the ways in which the pilot program was and was not working to improve care and communication among patients and providers. The focus group was digitally recorded by the interviewers and transcribed. Qualitative data, including meeting notes and observations, tracking data and the focus group transcript, were coded and organized by themes to focus findings and interpretation.

## Results

**Figure 1:**
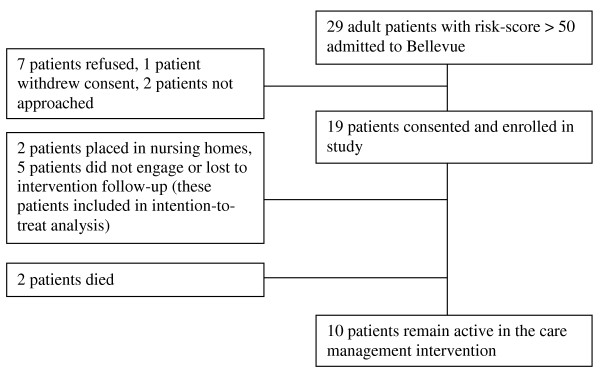
**Patient enrollment and participation**. This flow chart shows patients eligible for participation, those consented, and their study participation status.

During the study enrollment period, 29 Medicaid patients admitted to BHC had a high-risk algorithm score of over 50 and were eligible for the study. Seven patients declined participation, one patient provided consent then withdrew at enrollment, and two patients were not able to be reached by study staff during their admissions. The remaining 19 patients consented and were enrolled in the pilot study (Figure 1). Two patients who had been chronically street homeless prior to enrollment died within the first 12 months after enrollment. One died during the study enrollment admission due to complications from advanced alcohol-related liver disease, but fortunately our study staff was able to reunite him with family from whom he had been estranged. Another was diagnosed with metastatic cancer and died in hospice rather than on the street due to the work of our team. Because their deaths might have artificially created lower health services utilization than would have been the case if they had lived, both of these patients were excluded from this analysis.

Two additional chronically street homeless patients entered long term care facilities shortly after enrollment due to the work of our intervention team. Both were lucid and able to provide comprehensive histories at time of consent, yet, after more prolonged observation by our staff, we became concerned with the safety of both patients remaining homeless and their ability to safely care for themselves. Based on psychiatric and neurocognitive evaluations, both were subsequently placed in nursing homes and continued to be followed by our staff, yet were withdrawn from the study after discovering their diminished capacity. Our Institutional Review Board determined they could not be included in the final analysis.

Of the fifteen remaining patients, ten were actively engaged in the program, two were lost to follow-up, and three did not engage in program activities. The three patients who did not engage in the program had post-enrollment visits to our hospital, but despite repeated attempts at engagement by study staff, did not ever successfully receive pilot services. The two patients who were lost to follow-up never returned to our hospital or made contact with our study staff after enrollment despite our ongoing attempts.

All 19 enrolled patients were men (Table 1). The age range was 39-64 years old. Nearly all (18 of 19) were active substance users at the time of enrollment. The majority (15) used alcohol, 6 used opiates, and 2 used cocaine. Nearly all (17/19) were homeless or marginally housed. The two who were "housed" were in suboptimal living situations: one patient with severe pulmonary disease and disabling arthritis lived in a sixth floor walk-up apartment, and the other was evicted shortly after program enrollment and subsequently re-housed independent of our study. As shown in Tables 1 and 2, enrolled patients had multiple chronic medical conditions and substance use was linked to many admission diagnoses at study enrollment. The five patients who were lost to follow-up or did not engage in the program were younger (mean age 50) than those who did participate (mean age 57), and had slightly lower algorithmic risk scores (mean 64 compared to 70).

**Table 1:** Patient demographics at enrollment (n = 19)

Characteristic	Number (%)
Age, mean (range)	53 (39-64)
Gender	
Men	19 (100)
Race	
African-American	3 (16)
Hispanic	6 (32)
White	9 (47)
Mixed/Other	1 (5)
Substance Abuse	18 (95)
Homeless/Marginally Housed	17 (89)
Chronic Medical Conditions	
Hypertension	9 (47)
COPD/Asthma	7 (37)
Hepatitis B/C	7 (37)
Seizure Disorder	5 (26)
Coronary Artery Disease/Myocardial Infarction	4 (21)
Diabetes Mellitus	4 (21)
Skin Conditions (psoriasis, vitiligo, rash)	4 (21)
Deep Venous Thrombosis/Pulmonary Embolus	4 (21)
Gastritis/PUD/Esophagitis	3 (16)
Cirrhosis	2 (11)
Chronic Pancreatitis	2 (11)
Atrial Fibrillation	2 (11)
Hyperlipidemia	2 (11)
Lower Extremity Ulcers	2 (11)
Cancer	2 (11)

**Table 2:** Patient diagnoses at baseline hospital admission (n = 19)

Primary diagnosis	Number of patients
Chest pain	4
Alcohol Withdrawal	3
Cellulitis	2
Detoxification Services	2
Pneumonia/Bronchitis	2
Seizure*	2
Alcoholic Ketoacidosis	1
Asthma Exacerbation	1
Trauma (Fall)	1
Urinary Tract Infection	1

* Both patients admitted with seizure also had gastrointestinal bleeds at same admission

Eight of the total 19 enrolled patients met the federal Housing and Urban Development (HUD) criteria for chronic homelessness at enrollment (two years in a shelter, or one year on the streets or in a shelter with a disability) that determine housing eligibility in New York City. Of these eight, two were placed in nursing homes and two died, as mentioned previously. Due to a formal contract developed for this pilot, the remaining four patients were connected with and evaluated by our housing partner during their enrollment hospitalization, and were able to be placed in permanent housing.

**Figure 2:**
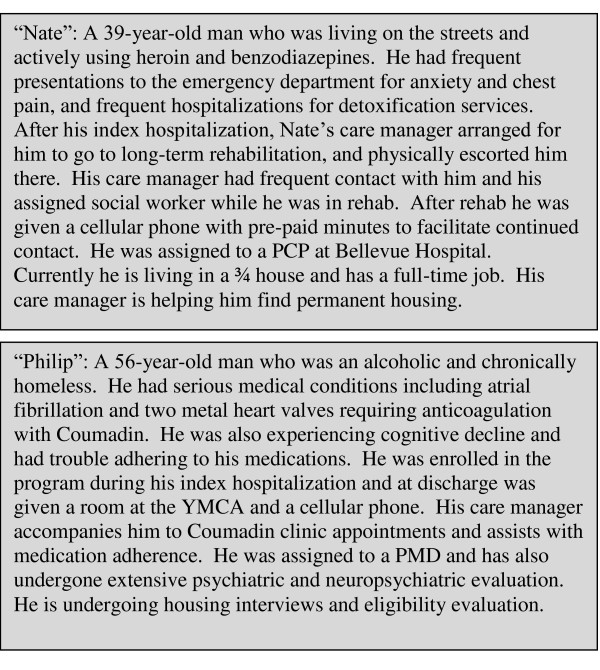
**Patient vignettes**. Two illustrative case examples of patients who participated in the pilot study.

Because our patients were each unique, we have provided two vignettes to illustrate some of the issues they faced and the resultant interventions (Figure 2).

### Care management

Patients received a substantial amount of care management time: on average, 11 hours per month. This time consisted of both direct patient contact as well as time spent coordinating and managing care. The mean number of contacts between patients and intervention staff was 17.6 per month (range 6-42). The average length of each contact was 30 minutes.

Interviews conducted with intervention staff revealed the need for relatively low patient to care manager ratios given the large commitment required per patient. Other challenges noted in interviews were the need to balance assisting patients with encouraging personal responsibility; the difficulty of changing patients' long-standing behaviors ("The problem is that they've had these patterns for so long. That even if you saw a change in six months, you're still in the beginning stages of change"); and the importance of addressing mental health and cognitive challenges ("...their physical conditions are so much more in their face and those aren't even being taken care of, so I think these underlying mental health issues, that they can kind of skate by and mask with substance abuse, are something that is our goal to address.") Staff additionally reported the expected bureaucratic issues that arise when interfacing with multiple City and community organizations, including fragmented service provision due to funding silos, and restrictive housing eligibility criteria.

Analysis of process data and focus group discussions suggest at least three core guiding principles necessary for working effectively with high-risk patients: flexibility, advocacy, and partnership. Our care manager spoke about letting go of expectations and straightforward linear treatment plans:

There is no ideal client. You catch people where they are and then provide them with what they need at that point. Doing that is always hard - I always say it's about catching them: you have to be mobile, you have to go where the ball is coming!

Staff also repeatedly described situations where they felt that they were the only advocates for their patients - that without them to translate, speak up for, or act on behalf of their patients, they would once again fall through the cracks. In addition, effective collaboration with all key service providers in patients' lives was essential:

It's important for us to develop relationships. Because [a social worker from an outside agency] is also serving one of our clients ...she'll kind of alert me now and call me and let me know what's up. You know, we've been trying to catch one of our clients at the methadone clinic. And getting that staff person there to agree to track [a patient] down and tell him to call us.

Relationships among team members were also invaluable for creatively solving problems, getting support to deal with frustrations, and sharing connections, resources, and strategies.

Lastly, using a non-judgmental, patient-centered approach was a critical ingredient in the intervention:

I do think how you approach patients makes a real difference from the patient perspective - because they're like wow here's a social worker or a case manager who is actually caring about me and I've had so many encounters with services where it was like "Bye! You're discharged." And I do think it's not just our persistence and our personalities but also our genuine care and concern.

A unique aspect of our pilot was the provision of cellular phones to enrollees in need. Most patients had no other way to stay in touch with staff, and both parties found the phones invaluable as a way to remain in communication. We provided a monthly pre-paid number of minutes and patients were instructed to use the phone mainly to keep in touch with staff or for related activities (e.g. calling for housing resources, to schedule job interviews). We did have limited incidents of lost and stolen phones. Despite these costs, we are continuing to provide cellular phones to patients in our current expanded intervention program, and believe staff and patients could not otherwise effectively maintain contact with one another.

### Health services utilization and costs

We used an intention-to-treat pre-post analysis, excluding the two patients who died during the intervention period and the two patients who were placed in nursing homes (all of these patients had fewer hospitalizations, emergency department visits, and clinic visits in the post-enrollment period).

**Figure 3:**
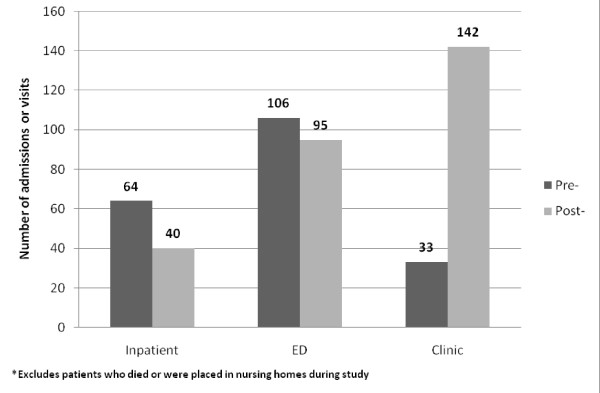
**Service utilization pre- and post-intervention**. This bar graph shows the number of hospital admissions, emergency department visits, and outpatient clinic visits made by study participants in the 12 months before and after the intervention.

Most (11 of 15, 73.3%) patients had fewer inpatient admissions in the 12 months after the intervention started compared with the previous 12 months (Table 3). The minority (4 of 15, 26.7%) had more hospital admissions after the intervention. The mean annual decrease per patient in admissions in the 12 months after enrollment was 1.6 (p = 0.18, CI: -0.83 to 4.03) and the median was 3. The total number of hospitalizations for all fifteen living, non-institutionalized participants decreased from 64 in the 12 months before the intervention to 40 in the following 12 months, for a 37.5% reduction in admissions (Figure 3). Overall, 106 emergency department visits were made in the 12 months before versus 95 emergency department visits in the 12 months after the intervention, a 10.4% reduction. The annual number of ED visits per patient was decreased by a mean of 0.7 visits, which was not statistically significant (p = 0.769, CI:-4.53 to 5.99). Eleven of 15 (73.3%) patients had fewer ED visits after enrollment.

**Table 3:** Individual patient health services use in the 12 months pre- and post-intervention*

	Hospitalizations		ED Visits		Clinic Visits	
	Pre-	Post-	Pre-	Post-	Pre-	Post-
**1**	4	5	14	10	2	17
**2**	2	1	5	1	20	39
**3**^**#**^	13	3	13	8	4	53
**4**^**=**^	5	1	5	4	0	1
**5**^**=**^	4	3	2	4	0	0
**6**	2	0	3	9	1	0
**7**	7	15	13	43	0	1
**8**	3	0	10	1	0	5
**9**	5	0	9	0	3	7
**10**^**^**^	3	0	3	0	0	0
**11**	3	0	6	0	0	0
**12**^**^**^	4	0	3	0	0	0
**13**	1	2	2	5	3	11
**14**	3	9	10	9	0	8
**15**^**=**^	5	1	8	1	0	0

* Excluding those who died or were placed in nursing homes

# High number of clinic visits for INR checks because patient on Coumadin

^ Lost to follow-up

= Did not engage

Outpatient clinic use varied by patient, with a statistically significant post-intervention increase of 7.2 visits per patient (p = 0.048, CI: 0.07 to 14.47). Much of this increase was driven by one patient on Coumadin with 53 post-intervention visits for INR checks; if he were excluded there was a somewhat smaller though still significant increase in post-intervention clinic visits of 4.3 per patient (p = 0.023, CI: 0.70 to 7.87). Most patients had the same (5 of 15, 33.3%) or more (9 of 15, 60.0%) outpatient clinic visits in the 12 months following intervention.

The decrease in inpatient hospital admissions after study enrollment resulted in a yearly per patient Medicaid cost reduction of $16,588 (p = 0.071, CI: -$1,613 to $34,788). The decrease in ED visits resulted in a $269 annual per patient reduction in Medicaid reimbursements (p = 0.065, CI: -$20 to $559), whereas the increased outpatient clinic use after the intervention resulted in an increase in per patient yearly Medicaid reimbursements of $474 (p = 0.106, CI: -$114 to $1,063) (Table 4). Considering inpatient, ED, and clinic costs together for all patients except those who died or were placed in nursing homes, total annual Medicaid reimbursements to the study hospital decreased by an average of $16,383 per patient per year (p = 0.073, CI: -$1,712 to $34,478). This represents a 38% reduction compared to the average annual Medicaid reimbursement of $42,996 per patient in the year prior to intervention enrollment.

**Table 4:** Average yearly Medicaid reimbursements per patient in 12 months pre- and 12 months post-intervention (n = 15)

	Pre-	Post-	Difference	p-value (CI)
Inpatient services	$42,696	$26,108	-$16,588	0.071 (-$34,788-$1,613)
Emergency Dept	$434	$165	-$269	0.065 (-$559-$20)
Outpatient clinic	$412	$886	+$474	0.106 (-$114-$1,063)

TOTAL	$43,542	$27,159	-$16,383	0.073 (-$34,478-$1,712)

* Excludes patients who died or were placed in nursing homes during the intervention period.

Total annualized intervention costs for this project totaled $169,551. Expenses included personnel support with fringe (0.25 FTE for the PI/medical director, 1 FTE for the Care Manager, 0.4 FTE for the Social Work Supervisor, 0.15 FTE for an administrator, and 0.10 FTE for IT/programming that was integral to our ability to conduct our daily match that facilitated patient enrollment) and Other Than Personnel Support (OTPS) costs, which included a "patient necessities fund" (for items such as urgently needed food and clothing that could not be obtained from in-kind sources), payment for transitional housing for patients awaiting permanent housing, mobile phone expenses, office supplies, travel expenses such as Metrocards (subway transportation) for patients, and computer costs.

We focused specifically on a population of high-risk, high cost Medicaid beneficiaries and were thus interested in the potential to reduce Medicaid spending. Subtracting our intervention costs ($169,551) from the total reduction in Medicaid spending ($245,745), the program resulted in a net reduction in Medicaid spending of $76,194. This represents an average annual Medicaid cost reduction of $5,080 per patient based on the 15 patients included in the analysis. This is likely a conservative estimate, as this is pilot work and our intervention costs are not necessarily to scale. For example, in the current expanded intervention program, per-patient intervention costs are markedly lower, as Care Mangers carry caseloads of 25.

Within this high-risk population, it is highly likely that patients will die or be placed in institutional settings after intervention enrollment. While our Institutional Review Board prohibited us from utilizing post-enrollment data for the two patients who were found to have limited decisional capacity and placed in nursing homes, we did conduct a separate analysis that included the two patients who died within the year after enrollment to take these outcomes into consideration. We found that including the patients who died in the analysis resulted in larger average decreases in hospitalizations and cost reductions. Total yearly hospitalizations in the study group (n = 17) fell from 72 pre-intervention to 41 post-intervention, and ED visits fell from 143 pre-intervention to 99 post-intervention. The patients who died after study enrollment had not made any pre- or post-intervention clinic visits, so these numbers did not change. Including the two patients who died, average yearly total (hospitalizations, ED, and clinic visit) Medicaid reimbursements per patient were $61,442 pre-intervention and $24,938 post-intervention. This amounted to a $36,523 average yearly reduction in Medicaid reimbursements per patient, which is more than double the $16,383 average reduction per patient when not including those who died in the analysis. Again, most of this reduction was driven by decreased costs related to hospitalizations. As is true for any study with a small sample size, overall results can be strongly influenced by extreme cases; one patient who died after study enrollment had a very expensive pre-intervention hospitalization ($301,744 in Medicaid reimbursements), which was a large contributor to the apparent improvement in cost savings when patients who died were included in the analysis.

## Discussion

We have described a pilot intervention for high risk, high-cost Medicaid patients that appeared to reduce overall inpatient hospitalizations. Our intervention was unique in its scope both within the hospital system and in the community, its robust collaboration with community based organizations, and its patient-centered approach. In the twelve months after the intervention started, patients had an average of 1.6 fewer hospitalizations per year and an overall reduction in Medicaid reimbursements of $16,383 per year. The pilot was associated with a trend towards reduced overall Medicaid spending when accounting for intervention costs. This is of key importance: predictive modeling in this population has shown that, without intervention, in the following year both the number of hospital admissions and costs to Medicaid would increase [1]. ED visits also decreased, while outpatient clinic visits increased. The overall decrease in hospital admissions was not statistically significant, which is not surprising due to the intervention's small sample size. We believe that the decreased number of readmissions to the hospital after the intervention was due in part to the fact that patients were receiving more effective outpatient care, and also to the assistance that our Care Managers provided to enrolled patients and staff in the ED that allowed some patients who would have otherwise been admitted to be discharged with follow-up from our intervention team. The bulk of reduced Medicaid spending in this pilot intervention resulted from a decrease in hospital admissions. This is in keeping with fee-for-service Medicaid's payment structure, which reimburses heavily for hospital admissions, and may differ for populations not insured by Medicaid.

Another important feature of the pilot intervention was our use of a previously validated case-finding algorithm to identify those Medicaid beneficiaries at most risk for future hospital admissions. Policy makers and researchers are increasingly aware that improving care management for the small group of very high-cost Medicaid beneficiaries presents one of the best opportunities for controlling costs [2,3]. Because using non-validated or informal techniques to identify patients for an intervention risks the misallocation of limited resources, other states, including Texas, Rhode Island, and Oklahoma, are attempting to use predictive modeling techniques to identify these highest-cost patients for intervention programs [4]. The case-finding algorithm used in the current study can be used by other hospitals or health systems as well, which would allow for targeted interventions to patients most likely to benefit.

Our program resulted in an average annual reduction in Medicaid spending of $5,080 per patient when accounting for pilot intervention costs. As mentioned, this is likely an underestimate for a few reasons. First, as this was pilot work, the program is not to scale. Based on cost projections for our recent project expansion which includes hundreds of patients, we believe this intervention will result in net Medicaid savings, even with a more modest annual reduction in hospitalizations, as our per patient costs are projected to be significantly lower, at approximately $3,500 per patient per year (versus pilot costs more than three times that amount). Developing an intervention that "pays for itself" is important given current budget deficits. Interventions that fail to result in savings are likely to be unpopular even if they do produce a significant human benefit.

It is important to bear the stakeholder in mind when accounting for health system savings. Inpatient admissions represent an important revenue stream for hospitals, so that while a reduction in admissions may lead to savings for Medicaid, this same reduction can result in decreased revenue for hospitals if the averted hospitalizations leave beds unfilled. However, it is not uncommon for high-risk patients such as those in this intervention to require prolonged inpatient stays--despite being medically stable--due to homelessness or other social factors that prevent a safe, prompt discharge. These prolonged stays can also lead to decreased hospital revenue as they are often not fully reimbursed and keep beds filled that might otherwise accommodate new admissions. Interventions such as this one may actually aid hospitals in being able to coordinate more efficient discharge planning. Also, as health care payment shifts from fee-for-service to potentially more efficient innovations including Accountable Care Organizations, the onus to improve care while reducing costs will increasingly be upon care providers rather than payers, motivating hospitals and other provider systems to find ways to decrease preventable hospital admissions.

There are several limitations to the current study. By design, it is a pilot study with a small number of patients. It is limited to one urban public hospital that may not be representative of other settings. Our enrollees were 100% male and were required to speak English or Spanish. We were unable to track study patients' visits to other hospitals, which may confound our results. Though many patients visited BHC preferentially, care managers observed that some "hospital shopped." However, this use of multiple hospitals would have presumably affected utilization frequencies both before and after the intervention. Costs were limited to those to which we had access from our study hospital, and do not include pharmacy costs. Pharmacy costs may have increased post intervention for patients who were previously non-adherent to medication regimens, or decreased for those who were previously (pre-intervention) receiving medications from multiple institutions or providers. For our expanded intervention, we will have access to Statewide Medicaid utilization data, which will enable us to accurately and completely track statewide healthcare utilization. The intervention costs listed are merely economic and do not address the potential for return to a higher functional state and therefore work or other societal benefits. It was also beyond the scope of this pilot to include all community costs (e.g. supportive housing) that will be important to achieve this type of intervention. Also, we did note that while hospitalizations decreased for the majority of pilot study patients after the intervention, there were four patients whose hospitalizations actually increased after the intervention. In our expanded intervention we will be able to better study what factors are associated with program success, which will enable us to target patients most likely to benefit and tailor program interventions accordingly in the future.

This pilot study was not a randomized control trial, thus it is possible that patient hospitalizations and ED visits fell for a reason other than our intervention. We feel this is unlikely, however, given the very strong effect observed and given prior research showing patients' costs are predicted to increase over time without intervention [1,13]. Finally, a few patients were lost to follow-up, which underscores the difficulties that can be encountered when working with a patient population marked by homelessness, substance abuse, and other social challenges. Despite these limitations, data from this pilot intervention offers useful information, especially to those looking to embark on similar projects.

## Conclusions

We have learned that an intervention with a very complex and high-risk patient group may be effective in reducing hospitalizations and Medicaid costs. High-risk patients require extensive contact with care managers that would not be otherwise achievable in busy ED or inpatient settings, but our data suggest that this time commitment pays off in outcomes. In addition, our partnerships with community based organizations were invaluable in allowing us to place several patients in permanent housing and provide other services not typically well-connected to the health care system.

The information gleaned from this pilot is currently being used to expand the program model across additional hospitals via a New York State Department of Health-sponsored Chronic Illness Demonstration Project. We are hopeful that the success of the pilot study can be replicated in this Demonstration, and plan a more detailed cost analysis as well as an analysis, using matched controls, of patient-centered health status outcomes such as self-rated health and patient satisfaction.

## Abbreviations

BHC: Bellevue Hospital Center.

## Competing interests

The authors declare that they have no relevant financial conflicts of interest to other competing interests. This study received approval by the Institutional Review Boards (IRBs) at the New York University School of Medicine and Bellevue Hospital Center.

## Authors' contributions

MR conceived of the study, and participated in its design and coordination, oversaw the data analysis, and helped to draft the manuscript. KD assisted with the data analysis and helped to draft the manuscript. BDE participated in the study design and portions of the data analysis, and helped to draft the manuscript. CG participated in the study design and coordination, and qualitative data analysis. SK participated in the study coordination and data collection. All authors read and approved the final manuscript.

## Pre-publication history

The pre-publication history for this paper can be accessed here:

http://www.biomedcentral.com/1472-6963/11/270/prepub
